# Pan-dent-emic: safety considerations for dental surgery in the era of COVID-19

**DOI:** 10.1186/s13037-021-00289-3

**Published:** 2021-04-12

**Authors:** Roma Enette Mascarenhas, Swati Pralhad, Nidhi Manaktala

**Affiliations:** 1 Department of Conservative Dentistry and Endodontics, Manipal College of Dental Sciences, Mangalore, Manipal Academy of Higher Education, Mangalore, Karnataka 575001 India; 2 Department of Periodontology, Manipal College of Dental Sciences, Mangalore, Manipal Academy of Higher Education, Mangalore, Karnataka 575001 India; 3 Department of Oral Pathology and Microbiology, Manipal College of Dental Sciences, Mangalore, Manipal Academy of Higher Education, Mangalore, Karnataka 575001 India

**Keywords:** Coronavirus, COVID-19, Dentistry, SARS- COV 2

## Abstract

The global coronavirus disease 2019 (COVID-19) pandemic spread has seized the entire world and has created extensive health concerns in the general population. Despite various efforts to prevent the pandemic spread, the flare-up of this disease is still soaring due to the community spread in every area. The droplet spread from the afflicted is of the highest concern because of its rapid spread to uninfected individuals. Dental treatments have to be planned and carried out with extreme caution and dental personnel should take extreme care and follow meticulous guidelines when treating an individual with SARS-CoV-2 (severe acute respiratory syndrome coronavirus 2) infection. This article highlights the clinical picture of COVID-19 (coronavirus diseases 2019) and presents a summary of precautionary and prophylactic measures in preventing the cross-infection and the nosocomial spread of the infection in a dental setting.

## Introduction

The aftermath of the COVID-19 pandemic has been unfathomable. It has affected every sector within health care industry with dentistry being one of the worst hits. Not only has it impacted the dental practice, there has been loss in terms of finances, psychology and most importantly breaks in ongoing education and research practices [1]. The COVID-19 infection was first identified in China when a cluster of pneumonia cases was reported in Wuhan [2]. Investigators found that these cases were infected by a previously unknown virus which they named 2019, SARS-COV-2 (severe acute respiratory syndrome coronavirus 2) infection. Despite strict measures taken to control the spread of the pandemic, the outbreak has exceedingly spread worldwide turning into a public health crisis. COVID-19 virus is a single-stranded RNA virus belonging to the family *Coronaviridae*. Their structure has a central core of genetic material abutted by casing of protein spikes at the periphery. This structure resembles the shape of crown, hence in Latin, the viruses get their name CORONA. The scientific name for COVID − 19 was given by the Coronavirus Study Group of the International Committee on Taxonomy of Viruses as SARS-CoV-2 [3].

There are divergent categories of COVID-19 viruses that give rise to gastric, enteric, and pulmonary prodromes. But the latest reports suggest that the corona viruses produce renal, neurological and digestive symptoms in few patients [4–6]. Respiratory symptoms can start with common cold and if left untreated, can lead to pneumonia which can be life-threatening. However, few of the corona virus species can increase the severity of the disease. These include the Severe Acute Respiratory Syndrome Corona Virus (SARS-COV) first noticed in China in 2003, and the Middle East Respiratory Syndrome Corona Virus (MERS-COV) first spotted in Saudi Arabia in 2012 [7]. The COVID-19 virus which was first invented in China, marked its inception in a boatload of people affected with pneumonia and was found to be linked with sea food or live animal market in the city of Wuhan. This nation’s malady was found to transmit via contact from the sick to others like family and healthcare workers. This disease spread at a rapid rate in China and was first thought to be an epidemic, but with the widespread overseas, it is now considered as a Pandemic [8, 9]. The main clinical characteristics of COVID-19 are not very different from SARS and MERS, with symptoms including cough, high fever, muscle pain, sneezing, respiratory tract infection, with about 7 to 10% of cases showing more serious complications evolving for pneumonia, and the reported lethality rate ranging from 1 to 2 to 3.4%, being significantly lower than that of SARS around 10% and that of MERS, around 30% [10].

The exact dynamics on the transmission of this virus is still unknown. In general, respiratory viruses get transmitted via droplet when an infected person coughs or sneezes or as a result of contact with contaminated surface. Based on the risk factors and the genomic spread of the virus, stern protective measures have to be taken for the prevention of the further spread of the disease.

### Clinical presentation of COVID-19 (SARS-COV-2)

The clinical presentation post infection from the virus are evident during or after the incubation period, which is 14 days and can extend for up to 23 days [11]. The infection manifest clinically as fever above 100 °C, shortness of breath, cough, fatigue, body aches. In occasional cases, reduced smell perception, altered taste sensation and headaches have been reported [12]. The symptoms range from mild to severe. In severe cases it leads to pneumonia, kidney failure and death. Chest X ray and Computed tomographic findings reveal bilateral ground glass opacities of the chest [11]. Strikingly, in majority of the population it can manifest as seasonal allergies and, flu-like symptoms develop which mimic the novel corona infection which are usually undetected making such people carriers, thereby increasing the number of persons infected [2]. However, though the number of deaths associated with the infection is high, the mortality rate is not very clearly understood. It has been noted that the mortality rate for persons aged 0–17 years is approximately 0.04–1%, 15–29 years it is 3%, 30–44 years it is 11%, 45–59 years around 32%, 60–74% around 39% and 75 years and above it is 14%. The mortality rate was seen to be more among the men (62%), than women (38.2%) [13]. However, the latest data shows that the mortality rate (India) has dropped down to 1.4% with a recovery rate of 97.32% as of February 17,2021 [14, 15].

### Viral transmission mechanisms in dentistry

**Fig. 1:**
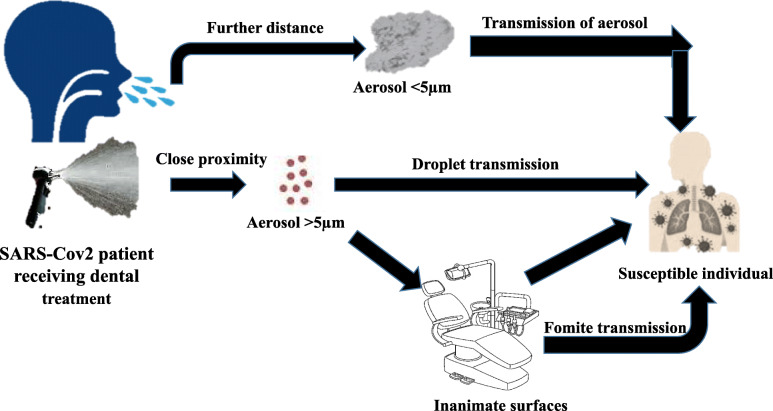
Schematic representation of spread of virus infection in dentistry

It is a respiratory virus, and hence the infection is known to spread via droplet or secretions. The respiratory and the salivary droplets from the infected individual can affect people in close acquaintance within a boundary of 6 feet. Thus, the implementation of social distancing and wearing of masks (N95 as advised by CDC) in public to prevent the rapid spread of the disease was elicited. The droplets of Corona virus on inanimate objects can be marked as another route of transmission. Therefore, disinfection of objects and handwashing regimen for 20 s has become the norm [10, 16]. Figure 1 shows the spread of the virus infection in dentistry.

The New York Times, on 15th March 2020 made a declaration in their publication that the dental fraternity are the highly risked personnel among the healthcare. As per the Occupational Safety and Health Administration (OSHA), dental health care personnel (DHCP) constitute high exposure risk category as they work in close proximity to the patient’s oral cavity. The aerosol generation from the dental instruments when treating patients is one of the key risk factor for the spread of the viral infection. With stringent infection control measures, the nosocomial spread can be prevented. Since the knowledge about the viral load and the spread is still growing, dental health care personnel have to undertake adequate measures to identify a suspected COVID-19 case and refer them to the specialized units for the treatment of the same [17].

### Diagnostic tests for COVID-19

The testing of COVID-19 virus is divided into three assays – Diagnostic testing, Serological testing and viral culturing. All of these have been developed by the Centre for Disease control and prevention (CDC). While the diagnostic testing include the (a) CDC Influenza SARS-CoV-2 Multiplex Assay and the (b) CDC 2019-nCoV RT-PCR Diagnostic Panel, the serological testing is done via the ELIZA assay to check for existence of antibodies, the specified proteins producing immune response to infections. Antibodies are investigated through blood tests from people after infection; to show body’s defenses to the infection [18].

### Proposed management strategies

During the unlock period there were various zones designated as hot-spots/red zones which were containment areas where Covid-19 positive cases were found in abundance Dental clinics were kept closed in the Containment Zone, however, tele- triage were provided for these patients. The patients from these zones were allowed to avail the ambulance services to visit nearby dental setup. Other zones like the orange and green zones were safer comparatively where dental practice was allowed in moderation. However, though the zoning doesn’t exist now; dental treatments have started full-on with complete protective measures treating each patient as an infected person.

**Fig. 2:**
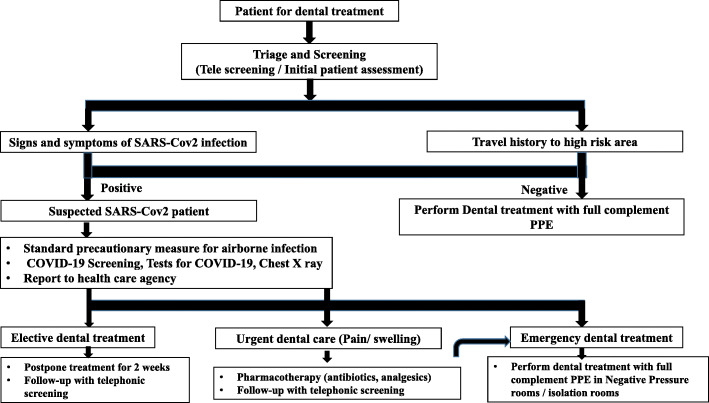
Flowchart based algorithm for treating patients during COVID-19 pandemic

Based on retrospective data of SARS–COV and COVID-19 viruses, specific guidelines have been set for the management of patients requiring dental treatment. These guidelines in India and across the world have been put forth by governing central and state dental regulatory bodies [18–22]. A decision-making algorithm for treatment of patients in dental clinics has been described in Fig. 2.

Dental management includes Pre-visit preparation, Dental office visit preparation and post-treatment management [22].

#### Pre-visit preparation-triage and screening/teledentistry

Sound knowledge of the spread of SARS-CoV-2 is required to prevent its transmission in the dental practice. Triage is the assignment to decide the order of treatment of a patient. Suspected COVID-19 patients can undergo screening via tele-triage. Initial triage should include relevant questionnaire such as any exposure to COVID-19 patients, any history of recent travel to foreign land or to any high-risk area of COVID-19, and any manifestations of pulmonary infections like fever, cough, and sore throat. To rule out high risk areas, global tracking system like Center for Systems Science and Engineering like those at Johns Hopkins University could be instituted. If any suspected individual shows a positive reaction to the questionnaire, the elective dental treatment should be postponed for at least 2 weeks [20, 21].

Pinto et al. recommend that the operative complex be divided into 5 zones. The zones include:

- Zone 1: Entry dressing room, where the basic PPE is donned.
- Zone 2: Anteroom, where the disinfection and surgical dressing take place
- Zone 3: OR (COVID-19 room)
- Zone 4: Exit room, where the PPE is removed
- Zone 5: Exit dressing room, where the staff showers

They suggested this route to minimize exposure and contact between triage to procedure room/OR (operating room) and then to recovery rooms and that they should be frequently cleaned and disinfected [23]. A similar pattern can be followed in a dental operatory to minimize the risk of infection.

#### Dental office visit preparation

##### Patient evaluation at the clinical setup

Upon the arrival of the patient at the reception counter of the dental set up, patient should provide personal details, complete details of medical history, questionnaire on COVID-19 screening, and the reason for true emergency. It should be made mandatory to check the patient’s body temperature using non-contact forehead thermometer. Patient who present with temperature above 100 ° F or 38°c, the dental treatment should be delayed for 2 weeks [21]. Patients should be seated in the reception area maintaining social distancing of about 6 ft. from one another. Aerosols are a predominant route for transmission of pathogens including SARS-CoV-2; therefore, stringent infection control measures are imperative [24]. Disinfection of hand using hand sanitizer should be made compulsory. Respiratory etiquette by wearing a surgical mask should be strictly followed. Patient should be requested to use a disposable tissue while sneezing and coughing and to discard it immediately. Any patient with suspected or confirmed SARS –COV-2 infection but in need of urgent dental care such as dental pain or swelling, should be strictly managed with pharmacotherapy in the form of antibiotics and analgesics. This may provide adequate time for the dentist to schedule an appointment for the treatment with full precautionary measures to prevent the spread of infection [21, 22].

##### Patient management

Philip F. Stahel divided elective procedures into “**essential**”, which bear an increased risk of adverse outcomes if surgery is delayed indefinitely, “**non-essential**” or “**discretionary**”, in which the results are not time-sensitive to surgery and “**equivocal**” which don’t fall clearly into one or the other category. He also projected a decision-making algorithm for deciding whether and when to proceed with an elective surgery, based on surgical indications and predicted requirement of critical resources [25]. Similarly, based on the urgency of the situation, a dentist can take a call so as to provide emergency treatment or to postpone the treatment. Emergency dental conditions based on the risk factors include uncontrolled bleeding, symptomatic pulpitis and periodontitis, tooth fracture/avulsion/luxation, facial fractures, facial space infections compromising airways and biopsies related to abnormal tissue [21, 22].

**Operative Examination of the patient**: Patients present in the clinical area for the examination should be set apart by at least one chair or 2 m distance. Care should be taken not to allow more than three patients in the clinical area. Operative examination should be done for only one patient at a time. Full complement personal protective equipment is recommended to be worn by the dentist. Centers for Disease Control and Prevention (CDC) has laid directions for donning and doffing of personal protective equipment when treating COVID-19 patients (https://www.cdc.gov/hai/pdfs/ppe/ppe-sequence.pdf) [21, 26]. Pre-procedural oral rinse with 1% Povidone iodine is recommended to lower the viral load of in saliva. It is recommended to perform extraoral swabbing prior to examination with isopropyl alcohol. Safe distance to be maintained during the examination of the patient. Informed consent should be obtained from every patient as a routine procedure. Every patient treatment procedure is performed with prior appointments so to provide ample time disinfection of the clinical area. Patient drape should be immediately discarded, and the examination chair should be disinfected with 0.01% Sodium hypochlorite [27]. It is highly recommended to use single use mouth mirrors, and probes, or the used instruments can be sprayed with ENZYMAX SPRAY GEL READY-TO-USE INSTRUMENT PRE-CLEANER to break the blood proteins and the bacteria and can be soaked in Septodont Quitanet Ultra or Glutapex solution for 15 mins to 20 mins.

**Guidelines/precautions for Treatment Procedure**: It is advisable to perform dental treatments in partitioned chambers to maintain safe distance and minimize contact. A modified concept of a “**corona-curtain**” in this regard, has been designed by Hill et al. where they discussed a simple, cost-effective, and innovative intubation tent designed to protect staff from viral aerosolization during emergent intubations [28]. Proper ventilation should be maintained with natural air by opening of windows and use of independent exhaust blower. Dental personnel (dentist and dental assistant) should wear the full complement personal protection equipment (PPE) during the treatment procedure. Every dental procedure should be performed under rubber dam with high volume evacuation to maintain the standard of care and to prevent splatter production. Extraoral HEPA (High efficiency Particulate air) suction with four handed dentistry can minimize the aerosol production [29]. The use of high-speed handpieces and ultra-sonics should be prevented. Micromotor handpieces with intermittent breaks and syringe for water irrigation is recommended as an alternative. Extraoral imaging such as Orthopantomograph (OPG) or Cone beam computed Tomograhy (CBCT) is better preferred over intra-oral imaging to prevent cross contamination. When the usage of intraoral imaging is must, it is advisable to cover the sensor with two barriers to prevent cross contamination [30]. Following treatment procedure. The patient drapes should be discarded immediately. It has been highly recommended to perform the treatment procedures in negative pressure treatment rooms or airborne infection isolation rooms [29]. It is mandated to maintain 1-h gap between the patient appointments so that the operatory can be disinfected and made readied for the next appointment. Data has shown that COVID-19/ SARS-COV-2 remain viable on inert surfaces for up to 3 days and has greater preference for humid condition [31]. It is recommended to vent off the air conditioning system in the operatory. In case of procedures involving use of general anesthesia (e.g. multiple extractions, fracture surgeries, rehabilitation treatment for pediatric patients etc.) a heat and moisture exchanger (HME) filter should be used on the expiratory limb of the circuit [32].. Dental impressions if taken should be disinfected by immersion in chlorine compounds, phenols, iodophors, formaldehyde and glutaraldehyde. Immersion in NaOCl at concentration of 1:10 (0.525%) is advised for 10 min [33]. The dentist/dental operating staff is recommended to perform five moments (Table 1) of hand hygiene recommended by World Health Organization (WHO) with Alcohol based hand rub (ABHR) [34]. These include:

- Moment 1. Before touching a patient
- Moment 2. Before a clean/aseptic procedure
- Moment 3. After body fluid exposure risk
- Moment 4. After touching a patient
- Moment 5. After touching patient’s surroundings

**Table 1:**
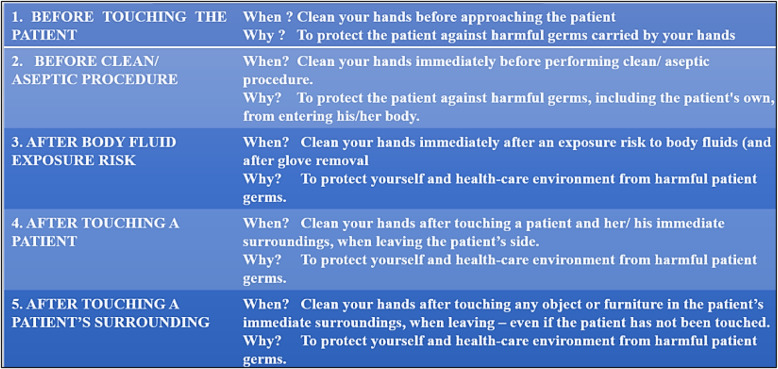
Five moments of hand hygiene recommended by the World Health Organization

#### Post-treatment/patient discharge protocol

After the treatment procedure, the assistant is advised to remove the drape from the patient and to discard it immediately. The patient is asked to perform hand washing and maintain the disinfection with Alcohol Rub. Then the patient is discharged from the operatory towards the reception area and requested to maintain social distancing. Post-operative instructions to be provided to the patient. Doffing of the PPE is done in the exit room.

##### Environment and surface disinfection and waste disposal

Environment and Surface disinfection should be performed with Totasep - Polyhexamethylene biguanide/Didecyl dimethyl ammonium chloride or 1% sodium hypochlorite with a contact time of 10 mins. Electronic equipment should be disinfected with 60–90% isopropyl alcohol. Suction system should be cleaned with Orotol or Aldexon daily at the beginning of the day to clear off the various microorganisms. Fumigation on a daily basis may or may not be practical for dental operatory; however, measures such as mopping the floor with 1% sodium hypochlorite and disinfecting waterlines with 0.01% sodium hypochlorite can help reduce the risk of cross infection [22].

##### Waste disposal

All the waste collected from the dental operatory should be considered as infectious waste and should follow the guidelines set by Central Pollution Control Board [22, 35].

**Table:** 

**Waste Disposal Protocol for COVID-19**	
• It is recommended to keep color coded bags and bins for proper segregation of waste.	
• As precautionary measure, double layering bags should be used for waste collection to ensure adequate strength and no perforation.	
• All the biomedical waste should be collected and stored separately and labeled as “COVID-19 WASTE” before handing over to an authorized staff of biomedical waste management.	
• The transportation of the waste from the isolation area should be done under PPE and recommend hand hygiene should be followed.	
• All the double layered bags have to be disinfected with 1% sodium hypochlorite as a routine protocol.	

## Discussion

The exposure of SARS-CoV-2 /COVID- 19 virus has put forth a lot of challenges to the dental fraternity. With the increasing numbers of COVID-19 cases, the dental healthcare is bound to treat patients with the new norm. Standard precautionary care while treating can reduce the risk of spread of cross contamination. Strict measures have to be ensured at every step of diagnosis and treatment planning in a dental set up. As per recommendation given by American Dental Association to institute only emergency treatment and to defer the elective treatment for a period of 2 weeks seem to continue for a longer period of time. The normalcy of treating patients would return once the vaccine for COVID-19 is found. This article highlights few suggestions on dental emergencies to provide better idea in treatment planning. Whether the dentist is an endodontist, pedodontist, prosthodontist, periodontist, an oral surgeon, any other specialist or for that matter a general dental practitioner; DHCP (dentists, dental hygienists, dental assistants, and receptionists) need to update their knowledge and skills regarding infection control and follow the protocols recommended by the relevant authorities to protect themselves and their patients against infections [22].

Dental fraternity should keep updating their knowledge on this pandemic virus and should train their staff to promote stringent infection control protocols in screening and treatment procedures. Dentists should consider every patient being treated as infectious and should take prompt measures in treatment procedures to prevent the spread of the infection. Despite the maximum measures taken, cases of re-infection have been reported. It was assumed that recovered SARS-COV-2 patients develop serum neutralizing antibody response to reinfection, but however few cases have shown that the antibodies developed wane off after few months. The correct theory behind the re-infection is still unknown and is under research. So, prompt standard of care precautions has to be taken while treating patients.

## Conclusion

Dentistry being a high risk profession, it is the duty of the health care staffs to maintain professional standard of care while treating patients in this pandemic situation. This review article highlights the need for high maintenance protocols in dentistry to protect the public from this novel virus. Thorough knowledge on aerosol generation, and its transmission should be updated at every stage to prevent any negligence during the treatment so as to prevent the rapid spread of the disease.

## Data Availability

Data sharing is not applicable to this article as no datasets were generated or analyzed during the current study.

## Abbreviations

SARS-COV: Severe acute respiratory syndrome coronavirus
MERS-COV: Middle Eastern Respiratory Syndrome coronavirus
SARS- COV 2: Severe acute respiratory syndrome coronavirus 2
COVID-19: Coronavirus Diseases 2019
WHO: World Health Organization
CDC: Centre for Disease control and prevention
PPE: Personal protective equipment
HEPA: High Efficiency Particulate Air
OPG: Ortho-Pantamo-gram
CBCT: Cone Beam Computed Tomography
ABHR: Alcohol based hand rub
IOPA: Intra-oral Peri-apical
HME: Heat and moisture exchange
DHCP: Dental Health care personnel
OSHA: Occupational Safety and Health Administration
